# Dynamic Variability of Isometric Action Tremor in Precision Pinching

**DOI:** 10.1155/2012/975735

**Published:** 2012-10-02

**Authors:** Tim Eakin, Waneen Spirduso, Karen L. Francis

**Affiliations:** ^1^Motor Behavior Laboratory, Department of Kinesiology and Health Education, The University of Texas at Austin, 1 University Station, Mail Stop D3700, Austin, TX 78712, USA; ^2^Department of Kinesiology, University of San Francisco, 2130 Fulton Street, San Francisco, CA 94117, USA

## Abstract

Evolutionary development of isometric force impulse frequencies, power, and the directional concordance of changes in oscillatory tremor during performance of a two-digit force regulation task was examined. Analyses compared a patient group having tremor confounding volitional force regulation with a control group having no neuropathological diagnosis. Dependent variables for tremor varied temporally and spatially, both within individual trials and across trials, across individuals, across groups, and between digits. Particularly striking findings were magnitude increases during approaches to cue markers and shifts in the concordance phase from pinching toward rigid sway patterns as the magnitude increased. Magnitudes were significantly different among trace line segments of the task and were characterized by differences in relative force required and by the task progress with respect to cue markers for beginning, reversing force change direction, or task termination. The main systematic differences occurred during cue marker approach and were independent of trial sequence order.

## 1. Introduction

The regulation of small force changes in hand digit musculature is of substantial interest to researchers in several fields because of its central role in the manipulation of objects and its importance in the ability to perform many of the activities of daily living. These volitional changes can be confounded by concurrent involuntary rhythmic force changes from tremor, early studies of which were primarily descriptive. However, recent development of specialized instrumentation has led to quantitative tremor analysis becoming a subject of wide research interest, see, for example, [1–3]. 

Among these newer investigative methods is the Manual Force Quantification System (MFQS), an instrumental system enabling quantitative tremor analysis in an isometric and goal-oriented task setting [4]. This system accommodates independent and simultaneous quantitative analysis of force impulse dynamics of two digits during pinch grip tasks, one of which is tracing a target line by independently varying the isometric forces applied by the thumb and index finger. Using the MFQS system, tremor contributions to aggregate force production can be extracted and studied independently from simultaneous volitional forces needed for task requirements [5]. 

Confounding tremor in fine motor control is generally of concern as a manifestation of action tremor, accompanying movement, but it also can occur in goal-oriented psychomotor control of isometric contractions. Such isometric tremor, being a periodic oscillatory pattern of force production changes, can be characterized by usual waveform variables such as frequency and amplitude. When multiple digits are involved in tasks requiring coordination of forces, variables can include phase differences between digit-specific tremor patterns. If a task is of short duration and static, such as in matching a constant force level, any confounding tremor is likely to be uniform, but for tasks of longer duration or which require regulated changes in force production, the tremor impact may vary within a single performance. Indeed, evidence exists that relative force level, for example, [6], and differences in force change direction, that is, application or release, for example, [7], influence the control of isometric force during steadiness [8] and during sine wave tracing using a single finger [9]. Task requirements of accuracy and inclusion of visually cued target goals can also impact dynamical organization of force production in a finger pinching task [10], which might also be a factor in the character of any concurrent tremor.

Thus, several questions about isometric action tremor in a goal-oriented task arise. The present study focuses on the confounding tremor force occurring in the Spirduso et al. [4] data set, analyzing it in more depth to ask the following questions: (1) are there significant differences in tremor characteristics that depend on the total tremor power present? (2) do dynamic differences exist in tremor characteristics that are related to changing levels of force required during a task? (3) do significant dynamic differences exist in tremor characteristics related to the pattern of volitional force changes during task performance? (4) does adaptation or adjustment occur that dampens tremor manifestation as a task progresses? and (5) does practice or fatigue impact tremor characteristics when repetitive trials are performed in a single testing session? To answer these questions we have developed a MATLAB program designed to analyze the Spirduso et al. [4] template tracing data by partitioning the original task tracing line into six segments characterized both by the relative force level required and by the task progress with respect to tracing cue markers. 

## 2. Materials and Methods 

### 2.1. Participants

Two groups were used for this study. One consisted of 10 individuals diagnosed with idiopathic Parkinson's disease (PD), mean age 63.1 ± 10.3 years. The other, serving as a control, consisted of 10 healthy older adults (OA) having no known neurological disorders, mean age 60.8 ± 9.8 years. All participants passed screening tests for vision, manual digit strength, medical stability and signed an informed consent affirming their willingness to participate. Those in the PD group completed a neurological examination and were rated on the motor section of the United Parkinson's Disease Rating Scale. Detailed participant demographics are given in Spirduso et al. [4] and all provided informed consent as approved by The University of Texas at Austin Institutional Review Board. 

### 2.2. Instrumentation

The MFQS apparatus used for designing tasks and measuring force production is described in detail in a previous paper [4]. Isometric force impulse magnitudes were collected at 100 Hz from two independent strain gauge transducers, mounted 180° with respect to each other, as a participant simultaneously applied or released pressure with the thumb and index finger of the same hand on the two transducer surfaces. The console was interfaced with a computer screen displaying a cursor whose horizontal and vertical positions were linearly related to force applied by the thumb and finger, respectively. A tracing template situated along a 45° line represented points of equal force from both digits, covering the range from 0.98 N to 4.45 N. At each end of the tracing line was a circular cue marker with a 0.098 N radius of acceptance indicating a boundary for a phase of the task. Specification of sampling rate, radii of acceptance for the cue markers, and location of the tracing line were parameters adjustable by the experimenter. All instrumentation settings and data acquisition procedures were controlled through a National Instruments DAQ board using LabVIEW.

### 2.3. Procedure

Each participant performed a set of 10 trials with each hand and each set was divided into two blocks of five, alternating blocks between hands. Hand order was counterbalanced for the OA group. The PD group started with the less affected hand, commencing one hour after-medication. 

The goal of the task was to trace the template line by increasing force equally from both digits between the start and return cue markers and then to similarly retrace back to the origin with controlled decrease of force. Participants were instructed to emphasize accuracy, keeping the cursor as close to the template line as possible, but to progress as quickly as possible while maintaining control. 

### 2.4. Data Analysis

Data from all successfully completed trials were used in the analyses. One trial each was aborted by two participants from the PD group and one from the OA group. Thus, less than 1% of the 400 attempted trials were omitted from the analyses.

Because the focus of this study was on variables associated with tremor, the first step performed in analyses of individual performance was the decoupling and isolation of the components of the aggregate force data which could be attributed to this source using the method of Eakin et al. [5]. Each FFT power spectrum of isolated tremor was scaled by the number of its samplings and for convenience in describing magnitudes the spectral power unit used was mN^2^ per sampling. A total power variable, characterizing tremor strength, was obtained by summing all thumb and finger power magnitudes at frequencies in the 5–7 Hz range. Similar to the logarithmically-based descriptions of seismic tremor magnitudes, a set of four levels of logarithmically spaced intervals was used to categorize the total power: background (<50); minimal (50–100); intermediate (100–1000); substantial (>1000). The frequency domain variables examined with respect to total power and its levels included the component sums of thumb and finger power, as well as the difference between them. Also examined were the particular frequencies at which the thumb and the finger power attained their respective maximal value and the difference between those frequencies.

One additional dependent variable was constructed from thumb and finger frequency amplitude vectors in the 5–7 Hz range after inverse FFT transformation back to the time domain, that is, the concordance phase. It is defined as the fraction of the sampling instances in which changes in force production by the two digits between sequential samplings are not changing in the same direction with respect to the laboratory frame of reference, multiplied by 180 in order to scale the measure identically with the phase angle scale [11]. A concordance phase of 0 represents a rigid sway where force production changes of the two digits are always in the same laboratory frame direction and a phase of 180 represents a repetitive pinching behaviour where the changes are always in the opposite laboratory frame direction. 

In order to confirm the occurrence of dynamic changes in tremor frequency during the course of a specific performance of the task, several individual trials in which the participant had exhibited noticeable tremor were partitioned temporally. The temporal partitioning was done by creating a 401-point moving window for samplings consisting of a centre point and 200 adjacent points on each side, for example, 1 through 401, 2 through 402, and so forth to (*N*-401) through *N* for a sequence of (*N*-401) sampling windows. 

**Figure 1:**
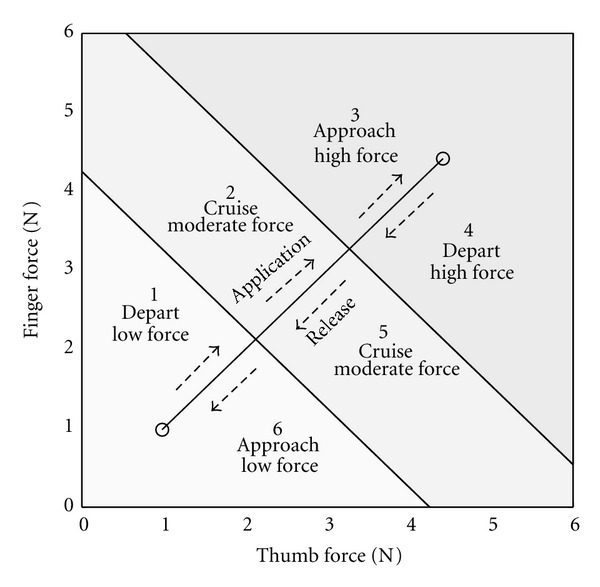
Segmentation of the tracing template. Lines perpendicular to the template divide it into thirds of equal length and define boundaries for categorization of relative task force level regions or task regions. The entire task is thus divided into six segments, three for tracing during application of force and three for tracing during release of force.

Similarly, in order to investigate changes related to requisite force level requirements and task regions, data from each task performance trial were partitioned spatially. For the spatial partitioning, the tracing template line was divided into thirds of equal length, and perpendicular lines (for equally scaled axes) were drawn through the division points to define boundaries for categorical aggregate force level and task objective segments. This partitioning created six task segments, three force application segments (1,2,3) for the first half of the task and three force release segments (4,5,6) for the remainder. A diagram is shown in Figure 1. 

Data samplings were grouped for statistical analysis in two ways based on this division. The first categorization was defined by the nearest point on the template line, with the aggregate thumb and finger force level assigned as low (segments 1 and 6), moderate (segments 2 and 5), and high (segments 3 and 4), though these labels were in a relative sense because all categories were “low” with respect to maximum voluntary contraction (MVC). The second categorization was task region, based on the tracing nature for the segment with respect to task requirement. Data samplings from segments 1 and 4 were considered to be in a region departing a visual cue marker, those from segments 2 and 5 were considered to be in a region of “cruising” between cue markers, and those from segments 3 and 6 were considered to be approaching a cue marker. 

Statistical evaluations were performed using SPSS software programs. In preparation for factorial repeated measures Multivariate Analysis of Variance (MANOVA) the data were aggregated by participant, creating mean scores for all variables over each of his or her 20 trials. Between-group tremor variable comparisons could be made because of the small amount of residual and background noise level of frequencies in the 5–7 Hz range that was present in even the steadiest of task performances by the OA group. This type of force production does not necessarily have origins related to the neuropathological source of PD tremor and the frequency distribution throughout the range was typically diffuse without the maximum power occurring within a well-defined peak.

A statistical significance level of *P* < 0.05 was used for between-group comparisons where tremor variables were considered collectively. However, when interrelated dependent variables (e.g., thumb-finger power difference, thumb-finger frequency difference) were being considered as concurrent factors, the significance level was subjected to a Bonferroni adjustment, leading to the use of a *P* < 0.01 criterion for rejecting null hypotheses. 

The questions under investigation all involve an examination of differences in tremor variable attributes according to within-participant levels of variable classifications and by between-group categories according to the PD and OA classifications. The analyses consisted of *N* × 2 repeated measures MANOVAs, where the particular within-participant designation was one having *N* categorical levels and the between-group distinction was always between PD and OA. Post hoc analyses were conducted in each instance to test for differences among the particular within-participant category levels. 

## 3. Results

**Figure 2:**
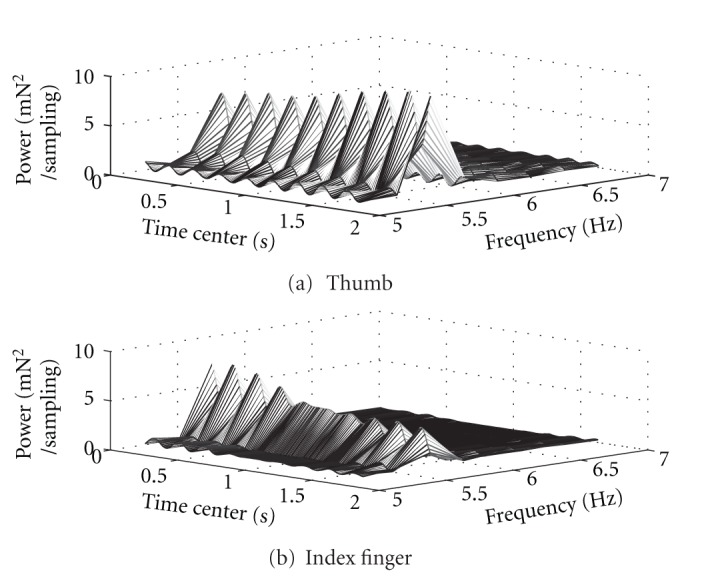
Example of the evolution of tremor power spectra. The three-dimensional surface plot shows the power spectrum in the 5–7 Hz range for isolated tremor during the force application phase of a trial performed by a PD patient using the more affected hand: (a) tremor from the thumb; (b) tremor from the index finger. Spectra were obtained from a moving four-second window, plotted sequentially with centres at 0.01 second intervals. Note that the peak power in this time region of the task appears oscillatory and the patterns for the thumb and for the index finger are distinguishable.

Trials performed by the PD group in which tremor power was at elevated levels and in which application or release involved intervals substantially more than four seconds provided data from which dynamic variation of within-task tremor variable values could be confirmed. A selection of such trials (including several participants, both hands, and both force change directions) was examined for changing patterns of power spectra as a series of 401 point (4 second) moving windows, incrementally offset according to the 100 Hz sampling rate. This did not reveal any particular uniform pattern descriptive of every trial, but the evolution of power spectra during these intervals exhibited a dynamic changing of power magnitudes within the 5–7 Hz frequency range. A surface plot of power spectra evolution for one particular force application interval is shown in Figure 2 to illustrate that the nature of tremor can rapidly change within the course of a single performance. 

**Figure 3:**
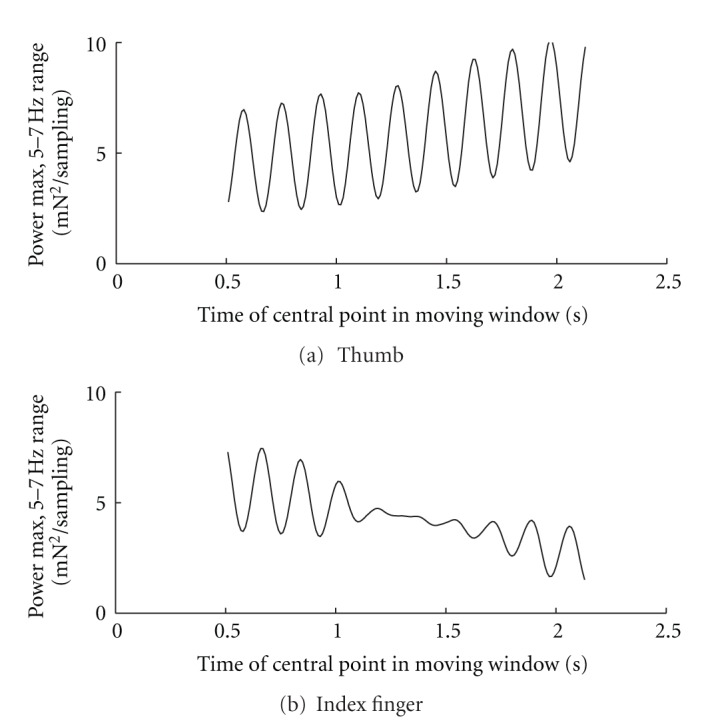
Example profile of peak tremor power fluctuations in the 5–7 Hz range. The data plotted represent the values from the crest of the peaks seen in the surface plot of Figure 2 as the 4-second moving window is advanced: (a) thumb peak power profile; (b) index finger peak profile. Notice that the regions of oscillatory behaviour are flanking a central region of less variable power magnitude for the index finger.

Though not a common feature in general it is nevertheless interesting that within this 5–7 Hz range not only was the power at the peak varying during the performance, but also the maximum amplitude showed an oscillatory magnitude as time progressed. This variability occurred for tremor in both the thumb and finger. The cross-sectional profiles of the power magnitudes at peak value, corresponding to the crest lines of the ridges in the surface plots of Figure 2, are shown in Figure 3. These plots show that evolution of tremor power in the thumb and finger is not necessarily parallel. Near the interval centre the finger peak power in this particular instance had relative stability while the thumb peak power continued to oscillate. Variable temporal patterns such as these, seen also in data from other trials with substantial levels of tremor power, prompted investigation of changes in tremor variable values based on both the overall magnitude of tremor itself and on intratask spatial considerations.

### 3.1. Grouping by Tremor Power

The two groups were not compared on task segments in the substantial tremor power category because none were present in the OA group performances. All other power levels (background, minimal, intermediate), which were present in both groups, were used for within-group factor comparisons.

A repeated measures MANOVA analysis using group as the between-subjects factor and tremor power level as the within-subjects factor revealed no main interaction between these factors. However, because the tremor power stratification was based on it, there was a significant within-group effect for power level [*F*_(12,30)_ = 7.129, *P* < 0.001, *η* ^2^ = 0.740]. Using univariate ANOVA tests, power level was confirmed as a significant factor for thumb, finger, and total power (all *P*'s <0.001; 0.592 ≤ *η* ^2^ ≤ 0.917). Likewise, power level was a factor for power and peak frequency differences, as well as for concordance phase [*F*_(1,2)_ = 9.16, *P* < 0.001, *η* ^2^ = 0.478]. Mean values are shown in Table 1. The effect on concordance phase is manifested as a decrease in value with increasing power level, particularly for the PD group. 

Thumb, finger, and total power means were significantly different from one another among the power levels, increasing as the level progressed from minimal to substantial (all *P*'s <0.001; 0.564 ≤ *η* ^2^ ≤ 0.923). The concordance phase values were also significantly different among power levels (all *P*'s <0.05; 0.402 ≤ *η* ^2^ ≤ 0.601). 

**Figure 4:**
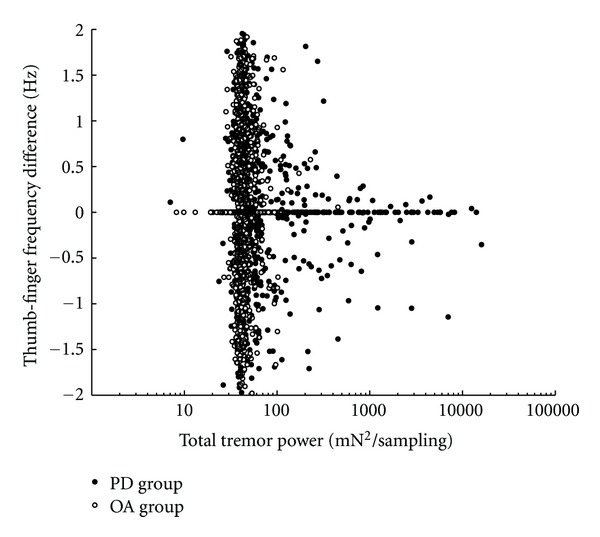
Scatter plot of the frequency difference variable against logarithmically scaled total tremor power using values from all trial segments of both the PD and OA groups. At background levels of total power the frequency differences are distributed throughout the theoretical [−2, +2] range, but the variance narrows around a mean near zero as the total tremor power increases beyond background.

**Figure 5:**
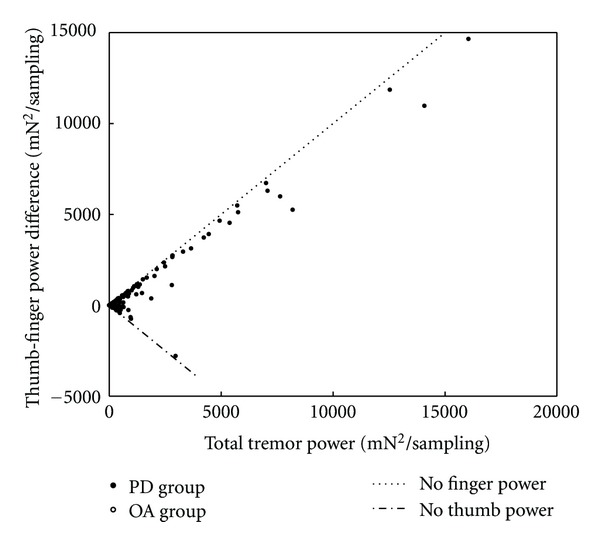
Scatter plot of the thumb-index finger power difference variable against total tremor power using values from all trial segments of both the PD and OA groups, although data from the OA group are too clustered for resolution of individual points. Limiting boundaries where all tremor power comes from a single digit are designated with dashed lines. As total tremor power increases beyond background level, the points remain close to the boundary lines, indicating a substantial asymmetry between the digits, with thumb dominance occurring in most instances.

**Figure 6:**
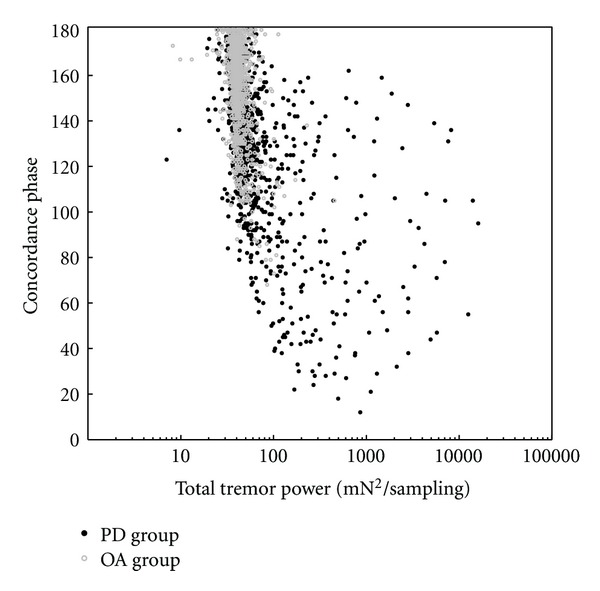
Scatter plot of the concordance phase variable against logarithmically scaled total tremor power using values from all trial segments of both the PD and OA groups, although data from the OA group are too clustered for resolution of most of those individual points. At background tremor power levels the concordance phase values are concentrated in the upper range of the scale, but as total power increases beyond the background level, many concordance phase values show up in the lower range of the scale.

In addition to the statistical analyses of the dependent variables in Table 1 based on power level, the distributions of their values as functions of the total tremor power were examined with scatter plots after pooling individual values from all six segments of every individual trial of all participants from both groups. A plot of the frequency difference distribution is shown in Figure 4. The variance narrows around a mean of zero as the total tremor power increases. A similar scatter plot of power difference values is shown in Figure 5. At higher total power values the data points are distributed close to the lines that demarcate boundaries where the entire power contribution would come from only one digit or the other. Thus the contribution to tremor from the individual digits is usually asymmetric as the total tremor power increases, with the thumb being the dominant contributor in most instances. Likewise a scatter plot of the concordance phase values is shown in Figure 6. Data in this figure from OA trials are clustered predominantly in the upper range of the concordance phase scale with little variance from the background total power around 40 mN^2^/sampling, thus resolution of most of the individual points is not possible. However, many data points from individual PD trials can be seen at higher total power values beyond background levels and they show dispersion into the lower range of the concordance phase scale.

### 3.2. Groupings by Task Region

Three separate groupings of the segments were considered: the relative amount of force required within a segment, the task progress with respect to the tracing cue markers, and the ordinal performance position of a segment within the task. 

#### 3.2.1. Analysis by Segment Force Level

Differences in tremor characteristics were examined when the six segments were collapsed into three force level categories (low, moderate, and high). The repeated measures MANOVA comparing the PD and OA groups revealed a main group effect when considering the tremor variables together [*F*_(5,14)_ = 3.43, *P* < 0.05, *η* ^2^ = 0.550]. Univariate ANOVA tests indicated that these group differences could be attributed to finger power [*F*_(1,18)_ = 14.49, *P* < 0.01, *η* ^2^ = 0.446] and concordance phase [*F*_(1,5)_ = 10.13, *P* < 0.01, *η* ^2^ = 0.360]. 

The repeated measures MANOVA also revealed a significant within-group effect for force level [*F*_(10,64)_ = 3.51, *P* < 0.01, *η* ^2^ = 0.355]. Univariate ANOVAs revealed that the main effect for force level could be attributed to total power and to differences in concordance phase (all *P*'s <0.01, *η* ^2^ = 0.233 and 0.288, resp.). Post hoc tests revealed that low force levels were different from moderate force levels for both total power and concordance phase; moderate force levels were different from high force levels for total power; and low force levels were different from high force levels for concordance phase (all *P*'s <0.05; 212 ≤ *η* ^2^ ≤ 0.455). No interactions were observed between group and force level. The means and standard deviations of variables contributing to the significant between-group and within-group differences are presented in Table 2.

#### 3.2.2. Analysis of Task Region Categories

The third question addressed differences in tremor variable mean values with respect to task region categories as displayed in Figure 1: departing (segments 1 and 4), cruising (segments 2 and 5), and approaching (segments 3 and 6). The repeated measures MANOVA revealed no significant difference between the PD and OA groups. However, there was a significant within-group effect for task region [*F*_(14,62)_ = 2.68, *P* < 0.01, *η* ^2^ = 0.385]. Univariate ANOVA tests indicated that task region differences are attributable to thumb, finger, and total power, and to concordance phase (all *P*'s <0.01; 0.241 ≤ *η* ^2^ ≤ 0.459). Results of post hoc tests were consistent with the PD group having substantially more tremor accompanying an approach to a cue marker than when cruising between markers or departing a marker. However, the variance relative to sample size was too great to assume a statistically significant group by task region interaction at the *P* < 0.05 level. Likewise, the concordance phase noticeably decreased for the PD group when approaching a cue marker. This variable also differentiated approaching from cruising and departing in the OA group, though the effect was not as great. The means and standard deviations of tremor power in the digits and of concordance phase, variables where significant differences are seen, are presented in Table 3. Post hoc analysis revealed that for thumb, finger, and total power, and for concordance phase, approaching a cue marker was significantly different from both departure and cruising regions (all *P*'s <0.05; 0.200 ≤ *η* ^2^ ≤ 0.635). No significant differences were found when directly comparing the departing and cruising regions.

#### 3.2.3. Analysis by Task Segment Ordering

The segmentation was also examined as a crude partitioning into individual task elements without any collapsing into categorical levels. The task was thus considered as six sequential parts without regard to force level or task region associated with those parts. This partitioning was sufficient to give a modest descriptive resolution in spatial groupings and in all but a few very fast performances and was sufficient to give a frequency resolution to at least the 1 Hz level, thus enabling investigation of the impact that specific combinations of task demand had on performance. The repeated measures MANOVA analysis comparing the PD and OA groups indicated significant differences when considering the tremor variables together [*F*_(5,14)_ = 3.42, *P* < 0.05, *η* ^2^ = 0.550]. Univariate ANOVA tests indicated that group differences could be attributed to finger power and concordance phase (all *P*'s <0.01; *η* ^2^ = 0.441 and 0.342, resp.).

Post hoc contrast analyses indicated that the between-group differences for both these factors could be attributed to several significant differences among the various segments. For the thumb power variable the only significant difference among the segments was that between the first (departing a cue marker at low force) and the fifth (cruising between cue markers during force release) segment. However, there were several significant differences in mean values of finger power and the concordance phase when comparing the various pairs of segments. These are summarized in Table 4.

The repeated measures MANOVA indicated significant differences for segment [*F*_(25,321)_ = 2.36, *P* < 0.001, *η* ^2^ = 0.118]. Univariate ANOVAs revealed that the main effect for segment was attributable to thumb, finger, and total power, and to concordance phase (all *P*'s <0.01; 0.178 ≤ *η* ^2^ ≤ 0.264).

Group and segment significantly interacted [*F*_(25,321)_ = 2.50, *P* < 0.001, *η* ^2^ = 0.124]. Univariate ANOVAs revealed that this interaction was attributable to finger frequency, finger power, and total power (all *P*'s <0.01; 0.197 ≤ *η* ^2^ ≤ 0.202).

### 3.3. Grouping by Trial Sequence Order

Lastly, influence of trial sequence order on tremor variable values, either from practice or fatigue effects, was examined. The repeated measures MANOVA analysis indicated a significant difference between the PD and OA groups when considering the tremor variables together [*F*_(5,14)_ = 3.42, *P* < 0.05, *η* ^2^ = 0.550], as had been found previously when collapsing across sequence order [4]. The univariate ANOVA tests indicated that group differences could be attributed to finger power and concordance phase (all *P*'s <0.01; *η* ^2^ = 0.443 and 0.338, resp.). However, no significant differences among trial sequence order within group were found and there was no group-by-trial sequence order interaction.

## 4. Discussion

Because the task for this study is isometric, the controlled application and release of force involve no movement by either digit. Nevertheless, the performance is not static and tremor variable values may change as a trial progresses both in time and in spatial representation on the monitor. The manifestation of such changes forms the basis for the questions being addressed here. 

Calculations from grouping of individual segments of trials by total tremor power give insight into the nature of tremor when it occurs at higher levels. This helps characterize PD tremor, but also provides clues about above-background tremor that was occasionally observed in the OA group. The PD group's trial segments having high tremor power, as well as the occasional ones of the OA group exhibiting noticeable power in the 5–7 Hz range, showed statistically significant differences in mean values of concordance phase relative to that at the background level. This supports the idea of a different oscillatory mechanism contributing to tremor power at background levels compared to that at higher levels and is consistent with the idea that the high power in the 5–7 Hz range which is seen occasionally in the OA group might have an origin similar to that causing PD tremor. It is also consistent with the finding of nonsynchronous force output for the thumb relative to the finger for various frequencies throughout the action tremor range during the holding phase of a multidigit grip task [12]. 

Other interesting observations from the PD group data arise when the total tremor power is used as an independent variable for other dependent variables. As the total tremor power of a task segment attains large values, there is a large asymmetry in the amount contributed by the two digits. Almost always the dominant digit is the thumb, which is usually the stronger of the two digits in terms of muscle mass. This may be more a reflection of a larger motor unit density in the thumb, though quantification of relative motor unit numbers in the two digits is not available. A second interesting observation is the distribution of the thumb-finger difference in frequency of maximum power in the 5–7 Hz range as the total tremor power within task segments rises above background levels. At the background level the values of those differences are located throughout the theoretical [−2, +2] range with approximately uniform distribution, but as tremor power increases the distribution narrows in variance around a mean value of zero. This would be expected if, at higher power, a common origin of tremor in the two digits was the predominant contribution while at background levels the frequencies of power maxima within the range was being determined randomly by fluctuations in a baseline.

To some extent the significant effects for total tremor power can be attributed to a relatively high level of volitional force required during the task (3.5 N). Because this is quite a small fraction of the typical MVC for older PD patients, the effect is not likely to be related to physical fatigue but rather may be an indirect consequence of the necessity for greater concentration at the higher force levels. It should also be noted that the high force level segment 3 involves cue marker approach, which can account for much of the difference that is observed.

The primary factor affecting tremor manifestation is the approach to a cue marker. This leads to an increase in the mean value of total tremor power and a decrease in the mean value of the concordance phase. It may be that extra mental stress or anxiety present when attending to coordination of volitional forces can trigger or amplify tremor, particularly in the PD group. Indeed, psychomotor tasks have frequently been characterized as those requiring “effort” (i.e., requiring more cognitive energy) compared to tasks requiring little or no “effort” [13, 14]. It is interesting that in a kinematical setting incorporating anisometric contractions a similar change in manifestation of force output upon approaching a defined target has been noted in PD patients [15]. However in that study the participants were on medication and tremor contribution to force output was not analyzed.

When performance is divided into spatially sequential task regions, the mean values of some dependent variables for tremor differ significantly among the various pairs of segments. However, the chronological ordering of the segments is not the determinant of any trend.

Because tremor is an involuntary phenomenon, it might be thought that dynamic changes in variable values would be stochastic fluctuations involving no trends or significant correlations and would not be affected by training. However, the tracing task does include elements of mental concentration and physical effort, so that mental and physiological factors such as anxiety, frustration, and fatigue could indirectly affect the manifestation of tremor during task performance.

The trial sequence order results obtained here were consistent with there being no significant training or adaptation effect on tremor manifestation from the repetition of trials during a testing session or from progressing through the task in the course of an individual trial. Each participant was tested in a single session lasting no more than a couple of hours; therefore, no conclusion about long-term training can be drawn. The lack of a significant effect from trial sequence order also applies to consideration of physical fatigue as a factor. Physical fatigue arising within a trial also should not be a significant factor in tremor manifestation because the maximum force production of each digit that is required by the task (3.5 N) should be no more than 5% of a participant's MVC, even for the elderly with PD.

Conclusions concerning statistical significance of temporal fluctuations of tremor variable values during the course of a trial cannot be drawn from these analyses because the task was not designed to have a uniform performance time, but general observations of power spectra from moving time windows show that dynamic changes in distributions of frequency amplitudes are a common phenomenon during performance. One interesting observation, though anecdotal, is the pulsatile nature of the maximum power profiles shown in Figure 3. Not only is the peak value changing during the course of the trial, but also changing in an oscillatory pattern of its own, and with a frequency similar to the frequency of the underlying tremor itself. 

### 4.1. Limitations

The major limitations in interpreting data stem from the necessity of evaluating what are essentially continuous variables using discrete methods. This primarily impacts variables related to frequency in both individual digits and in their difference. The FFT algorithm procedure limits the number of component frequencies to the number of elements in the input vector and distributes them only with equal spacing in the range up to the data acquisition rate. Furthermore, half the FFT information is redundant because of symmetry properties. This can create problems for analysis of trial segments traversed too fast to accommodate enough frequencies within the range of interest. Power for a true natural frequency could also be dispersed among the algorithmically generated component spectral frequencies. For reasons such as these, values calculated for frequency-based tremor variables should be interpreted with caution. These sorts of limitations are commonly found whenever simultaneous high resolution in both a spatial domain and in a frequency domain is desired. The partitioning used in these studies has been chosen as a compromise in this tradeoff of competing spatial and frequency resolution.

## 5. Conclusion

Bearing in mind the limitations, this study nevertheless has shown that force production from nonvolitional tremor can have significant temporal and spatial variation within the course of a single task trial. In particular, the level of volitional force required by the task and the process of guiding a cursor to approach a cue marker affect tremor characteristics. Substantial information can thus be overlooked if system variable values are derived as single averages over the entire ensemble of sampling points. 

## Figures and Tables

**Table 1:** Means and standard deviations of tremor variables stratified by total tremor power level.

Grouping level^a^	*n* ^b^	Frequency difference (Hz)^c^	Power difference^c,d^	Concordance phase
PD background	194	0.019 (0.162)	0 (3)	145 (11)
PD minimal	90	− 0.018 (0.247)	−1 (6)	126 (19)
PD intermediate	86	0.072 (0.278)	38 (122)	104 (34)
PD substantial	26	− 0.087 (0.127)	2289 (3090)	92 (34)

OA background	354	0.044 (0.092)	0 (1)	154 (17)
OA minimal	41	0.034 (0.334)	0 (1)	154 (17)
OA intermediate	3	0.233 (0.273)	36 (42)	107 (31)
OA substantial	0	—	—	—

PD: Parkinson's disease participants.

OA: older well adults.

^a^Based on total tremor power (sum of thumb and index finger) in mN^2^/sampling.

0–50: background; 50–100: minimal; 100–1000: intermediate; 1000+: substantial.

^b^Total number of application and release task segments in the power grouping.

^c^Differences represent index finger values subtracted from thumb values within trials.

^d^Power difference units in mN^2^/sampling.

**Table 2:** Means and standard deviations of tremor variables stratified by requisite task force level.

Task-relative force levels^b^	Finger power^a^		Total power^a^		Concordance phase	
	PD^c^	OA^d^	PD	OA	PD	OA
Low	52 (86)	24 (12)	229 (957)	48 (27)	130 (34)	152 (22)
Moderate	27 (30)*	21 (4)	96 (368)	41 (7)	136 (33)*	157 (19)
High	54 (198)	20 (3)	281 (1237)*	41 (7)	133 (36)	155 (19)

PD: Parkinson's disease participants.

OA: older well adults.

*Significant.

^a^Power units are in mN^2^/sampling.

^b^Magnitude of thumb and index finger force level required to move the cursor through each task segment.

^c^Total number of application and release task segments (PD group, *n* = 396).

^d^Total number of application and release task segments (OA group, *n* = 398).

**Table 3:** Means and standard deviations of tremor variables stratified by task region with respect to cue marker objective.

Grouping^a^	*n* ^b^	Thumb power^c^	Index Finger power^c^	Concordance phase
PD departing	396	61 (100)	30 (14)	137 (21)
PD cruising	396	69 (81)	27 (6)	136 (18)
PD approaching	396	345 (437)*	76 (52)	127 (20)*

OA departing	398	22 (3)	21 (1)	157 (11)
OA cruising	398	21 (1)	21 (1)	157 (11)
OA approaching	398	23 (4)	22 (3)	150 (12)*

PD: Parkinson's disease participants.

OA: older well adults.

*Significant.

^a^Based on task segment region with respect to cue marker objective.

^b^Total number of application and release task segments in the grouping.

^c^Power units are in mN^2^/sampling.

**Table 4:** Significant differences (*P* < 0.05) in paired segment variable means from post hoc analyses.

Segment			1	2	3	4	5	6
				Apply			Release	
			Low force depart	Mod force cruise	High force approach	High force depart	Mod force cruise	Low force approach
1		Low force Depart	~~~	F		F	F	
2	Apply	Mod force Cruise	C	~~~	F			F
3		High force Approach	C	C	~~~	F	F	

4		High force Depart			C	~~~	F	F
5	Release	Mod force Cruise					~~~	F
6		Low force Approach	C	C	C	C	C	~~~

F: significant difference in means of index finger power (upper right).

C: significant difference in means of concordance phase (lower left).
